# Unnecessary dental extractions in patients with Persistent Idiopathic Facial Pain: a qualitative and questionnaire-based study of patient perspectives

**DOI:** 10.22514/jofph.2026.016

**Published:** 2026-03-12

**Authors:** Dror Shir, Iftah Biran, Rakefet Zalashik, Amnon Mosek

**Affiliations:** ^1^Cognitive Neurology Unit, Tel Aviv Sourasky Medical Center, 6423906 Tel Aviv, Israel; ^2^Division of Psychiatry, Tel Aviv Sourasky Medical Center, 6423906 Tel Aviv, Israel; ^3^School of Public Health, Ben-Gurion University of the Negev, 8410501 Beer Sheva, Israel; ^4^Headache and Facial Pain Clinic, Department of Neurology, Tel Aviv Sourasky Medical Center, 6423906 Tel Aviv, Israel

**Keywords:** Persistent Idiopathic Facial Pain, Chronic pain, Qualitative research, Somatization

## Abstract

**Background**: This study aimed to explore the perspectives of individuals with Persistent Idiopathic Facial Pain (PIFP) who sought dental extractions for pain relief and to identify common themes from their experiences. PIFP significantly impacts the quality of life, leading many patients to undergo unnecessary dental procedures on healthy teeth for pain relief. Recognizing unique characteristics in patients with a history of such interventions could help prevent unnecessary treatments and associated complications. **Methods**: We conducted qualitative research at the Headache Clinic, Tel Aviv Sourasky Medical Center, involving 12 consecutive patients with PIFP who had undergone dental extractions. Data were collected through medical records, interviews, and questionnaires. The recorded interviews were transcribed and analyzed using qualitative research guidelines, with a focus on descriptive, linguistic, and conceptual comments. **Results**: Twelve participants aged 28–83 were included in the study. Data analysis revealed three main themes: (1) physical metaphors (“like an exposed nerve”), (2) emotional and cognitive reactions to pain (“life had stopped”), and (3) encounters with the medical establishment (“not just injustice, it’s medical negligence”). Physical metaphors included additional somatization, symbolic penetration, facial pain analogous to emotional pain or a traumatic event, and pain as a silencer. Emotional and cognitive reactions included catastrophic reactions, incomprehensibility, loss of agency, and disconnection from emotional pain. Finally, encounters with the medical establishment included complex interactions with medical figures, as well as confusion and perplexity with the medical system. **Conclusions**: This qualitative study offers insights into the subjective experiences of PIFP patients. The identified themes highlight shared challenges and the multifaceted nature of PIFP, underscoring the need for comprehensive, multidisciplinary approaches.

## 1. Introduction

Persistent Idiopathic Facial Pain (PIFP), formerly known as atypical facial pain, is marked by persistent, dull facial and/or oral pain without a neurologic deficit [1]. While the incidence of PIFP is 39.5 per 100,000 person-years [2], estimates of prevalence vary widely [3]. The pain, often described as poorly localized, dull, or aching, may affect areas such as the nasolabial fold or one side of the chin but can extend to broader facial and neck regions. This pain may follow minor surgical procedures or injuries to the face, teeth, or gums and persists despite physical healing, lacking a clear local cause and not adhering to a peripheral nerve distribution [1, 4, 5]. According to the International Classification of Headache Disorders 3rd edition (ICHD-3), the diagnosis of PIFP requires daily pain for more than two hours per day over at least three months, a normal neurologic examination, and exclusion of dental causes [1, 6].

The role of dental extractions in PIFP remains controversial, with unclear implications for the disorder’s pathogenesis [7, 8]. Reports indicate that 99.3% of PIFP patients initially consulted a dentist, and 83% underwent dental interventions. However, 67% still experienced pain post-treatment [9, 10]. Persistent pain may also lead to psychological distress, limited reversibility, and additional postoperative complications.

Despite the lack of strong evidence, PIFP is sometimes linked to psychogenic origins [11]. Due to the elusive nature of idiopathic facial pain, patients often encounter a lack of understanding and support from medical professionals [12, 13]. There is a knowledge gap, as prior research predominantly focused on diagnosis, pharmacological interventions, and epidemiology, with limited attention to patients’ subjective experiences and the psychological factors influencing their clinical presentation [14, 15].

Within the broader field of chronic orofacial pain, PIFP represents a distinct and understudied condition. Its enigmatic nature, lack of identifiable pathology, and frequent association with unnecessary dental procedures make it a critical focus for understanding patients’ subjective experiences beyond biomedical explanations. Patients with PIFP frequently use physical metaphors to describe their pain, offering insights that could improve pain management strategies and support [16, 17, 18]. To date, no qualitative studies have specifically explored the narratives of PIFP patients. Lovette *et al*. [12] examined Chronic Oral Facial Pain (COFP), a category that includes PIFP, and identified three thematic clusters (biomedical, psychological, and social), though only 14 out of 260 patients (5.4%) had PIFP [19].

Following Schweiger *et al*. [7] and based on our clinical experience, we suspect that the broad PIFP definition encompasses a heterogeneous group of pain symptoms, with those seeking dental extractions forming a distinct subgroup that merits special attention.

This study aims to identify and describe the primary clinical features and prevailing themes expressed by individuals in this subgroup within a tertiary care center specializing in facial pain and headache. Drawing on Munday *et al*. [20], who highlighted the interpretive value of pain metaphors (causal, experiential, bodily, and death-related), our study explores the perspectives of patients with PIFP who underwent dental extractions for pain relief. We employed semi-structured interviews with individuals diagnosed with PIFP to gain an in-depth understanding of the subjective experience, addressing the gap in the existing literature, which mainly focuses on the biological dimension of the disorder and less on the patients’ psyche and subjective experience [14]. By using semi-structured interviews, we aimed to understand PIFP beyond the biomedical model and to examine how these patients manage their pain and construct their clinical realities in the absence of an identifiable cause. This qualitative approach, complemented by self-report questionnaires, enables a deeper understanding of the psychological and clinical dimensions underlying patients’ experiences [20].

## 2. Methods

### 2.1 Semi-structured interviews

We conducted semi-structured interviews with individuals diagnosed with PIFP to gain an in-depth understanding of their subjective experiences. PIFP is characterized by poorly localized pain, which is described as “elusive in nature” [1], making semi-structured interviews uniquely suited for an in-depth exploration of individual perceptions. This patient-centered qualitative approach enables the uncovering of nuanced insights and rich narratives of PIFP patients who often report a lack of understanding from medical professionals [12].

### 2.2 Self-report questionnaires

In addition to the qualitative interviews, participants completed a battery of clinical self-report questionnaires, all described in section 2.4, to gather quantitative data on various psychological and health-related dimensions. We were especially interested in the following dimensions: depression, anxiety, somatization, dissociation, illness perception, as well as mental and physical well-being. Despite the limitations of self-report measures [21], their inclusion enabled a comprehensive assessment of participants’ psychological and health status, complementing the qualitative interview data.

### 2.3 Participants

Participants were adults diagnosed with Persistent Idiopathic Facial Pain (PIFP) who had undergone one or more dental extractions, continued to experience PIFP symptoms, and were proficient in Hebrew. Recruitment was conducted through the Headache Clinic at Tel Aviv Medical Center between 2019 and 2020. All active patients in the clinic who met the ICHD-3 and International Classification of Orofacial Pain (ICOP) diagnostic criteria for PIFP were considered [1, 22]. By definition, neurological examination was normal, with no abnormalities of the facial nerves or muscles. As quantitative sensory testing was not performed, the ICOP code assigned to all patients was 6.2—Persistent idiopathic facial pain (general category, Quantitative Sensory Testing (QST) not performed). Of the sixty-six subjects who met these criteria, twenty-four had undergone at least one dental extraction as a treatment attempt. Twenty of the twenty-four patients were successfully contacted; among them, three no longer had active pain, one was excluded due to a language barrier, and four declined participation. The remaining twelve patients were interviewed and included in the study (see Fig. 1 for the flowchart of the participant recruitment process).

**Fig. 1.** Flow chart of participant recruitment process. PIFP: Persistent Idiopathic Facial Pain.

### 2.4 Procedure

Participants were interviewed on one occasion in the headache clinic or at their own homes, with one participant interviewed via Zoom, between 2019 and 2020. All qualitative, semi-structured interviews, lasting between 60 and 100 minutes each, were consistently administered by the same interviewer (RZ). These interviews comprised open-ended questions tailored to the research objectives. Questions included characterization and localization of the pain, disease duration and timeline, major life and medical events, events occurring before symptom onset, effects on daily life and well-being, and the impact of living with chronic facial pain.

After the interview, participants completed self-report questionnaires, which took approximately 30–90 minutes. They were asked to fill in any missing items and were encouraged to take breaks as needed to maintain comfort and data quality.

The questionnaires included the following: The Brief Symptom Inventory (BSI) [23]—53 items across nine symptom dimensions, with *Z*-scores >2 considered abnormal [24]. Hospital Anxiety and Depression Scale (HADS)—14 items assessing anxiety and depression; scores >10 indicate clinical concern [25, 26]. The Brief Illness Perception Questionnaire (BIPQ)—9 items (8 quantitative, 1 qualitative); total score >42 denotes a significant perceived threat [27, 28, 29]. SF-36 (The Short Form Health Survey)—measures physical (Physical Component Summary (PCS)) and mental (Mental Component Summary (MCS)) health; mean = 50, higher scores = better status [19, 30, 31]. The Somatic Dissociation Questionnaire (SDQ)—20 items on somatic and dissociative symptoms; scores >30 suggest somatoform disorder [32, 33, 34]. The Hebrew Dissociative Experience Scale (HDES)—28 items assessing dissociative experiences; cutoff 45–55 differentiates clinical from normal [35, 36]. For analysis, the following summary scores were used: BSI–Somatization, BSI—Global Severity Index, SDQ—Total Score, HADS—Total Score, BIPQ—Total Score, SF-36—MCS, SF-36—PCS, and HDES—Total Score.

### 2.5 Data analysis

Interviews were transcribed verbatim. We used a multistage thematic synthesis approach; The final set of themes was rigorously reviewed for inter-rater reliability by two independent authors (DS and RZ) to ensure trustworthiness in the coding process. The transcripts were read and re-read, and the authors made descriptive, linguistic, and conceptual comments. To ensure qualitative validity, the lead researcher maintained a detailed log of reflective comments to monitor how pre-existing clinical assumptions influenced the analysis. The authors identified and categorized twenty-five recurring themes in the interview analysis process, selecting quotes from each interview. Data collection continued until theme saturation was achieved, with no new conceptual insights emerging from the final interviews. Subsequently, emergent themes were clustered into subthemes and then synthesized into three overarching categories: (1) physical metaphors, (2) emotional and cognitive reactions to pain, and (3) experiences with medical care and systemic confusion [37].

## 3. Results

### 3.1 Patient characteristics

The study included ten women and two men who met ICHD-3 criteria for PIFP. Demographics and patients’ characteristics are shown in Table 1. Participants’ ages varied greatly (median range, 49 [28–83]). Mean illness duration was 4.3 ± 3.3 years. The mean number of teeth extractions per patient was 2.9 ± 2.4 teeth. A history of additional somatization was observed in 8 out of 12 patients (Table 1). Pain locations were mapped according to patients’ reports (Fig. 2). The most frequently reported sites for localized pain were specific teeth (in 4 out of 12 patients) or the upper/lower jaw (in 4 out of 12 patients).

**Table 1.** Participants’ demographics.

Patient num.	Age, (yr)	Sex	Profession	Illness duration, (yr)	Number of teeth extracted	Other areas affected by unexplained pain	Pain management history
1	28	F	Student	1	2	Stomach aches	Amitriptyline, Carbamazepine
2	83	F	Retired	10	4	“Heart blockage”	Amitriptyline, Gabapentin, Carbamazepine, Clomipramine, Duloxetine, Escitalopram, Pregabalin
3	43	F	Travel Agent	1	1	Stomach aches	Carbamazepine, Steroids
4	69	M	Retired	1	4	Chest pain, radiating and wandering diffuse pain	Amitriptyline, Diazepam, Dipyrone, Etodolac, Sertraline
5	56	F	Unemployed	3	9	Oral ulcers, diffuse pain	Amitriptyline, Gabapentin, Pregabalin
6	57	F	Coordinator in non-profit organization	9	1	Fibromyalgia, “electrical currents in the head”	Amitriptyline, Botulinum Toxin, Cannabinoids, Duloxetine, Erenumab-aooe, Milnacipran, Pregabalin
7	40	F	Attorney	1	1	-	Pregabalin
8	38	M	Teacher’s assistant	3	3	Chronic backaches	-
9	40	F	Secretary	8	3	-	Amitriptyline
10	28	F	Actress	6	1	-	Acetyl Salicylic Acid, Amitriptyline, Oxycodone, Propranolol, Rizatriptan, Topiramate
11	79	F	Retired	5	5	-	Amitriptyline
12	76	F	Retired	4	1	“Inflammation in the intestines”	Amitriptyline, Carbamazepine, Clomipramine, Clonazepam, Duloxetine, Ibuprofen, Naproxen, Oxycodone, Pregabalin, Sertraline, Sulpiride
Total*	53 ± 19	F = 10 (83%)	-	4.3 ± 3.3	2.9 ± 2.4	-	

Participants characteristics table. *Mean ± SD for continuous variables, count (%) for categorical variables. F: Female; M: Male; SD: Standard Deviation; num: Number.

**Fig. 2.** Facial pain locations across study participants. P: Patient; TMJ: temporomandibular joint.

### 3.2 Questionnaires

Table 2 summarizes the scores on the questionnaires and responses to the open-ended question of the BIPQ. The results indicate varying levels of somatic dissociation, psychological distress, illness perception, and health status among the patients. The mean score on the SDQ was 26.25 (Standard Deviation (SD) = 4.54). Although the mean score was below the somatoform disorder threshold of 30, individual scores ranged from 22 to 36, with two patients exceeding and three approaching the cutoff, suggesting somatoform tendencies in some participants. The mean somatization score on the BSI was 0.65 (SD = 0.98), with most scores falling within the normal range. However, one patient exhibited an abnormal score above the cutoff (*Z* > 2), indicating elevated somatic symptoms. The BSI General Severity Index (GSI) had a mean score of 0.27 (SD = 0.82), also indicating mild overall psychological distress. The mean HDES score was 6.15 (SD = 6.15), which suggests that, overall, the cohort did not exhibit severe dissociative symptoms. The BIPQ mean score was 58.33 (SD = 9.00), reflecting a perception of high experienced illness severity. The most frequent response (7/12) to the open-ended question of the BIPQ (*e.g.*, please list the most important factors that you believe caused your illness) attributed the facial pain to physicians, dentists, or dental procedures. The mean HADS score was 17.17 (SD = 7.76), indicating mild to moderate symptoms of anxiety and/or depression across the cohort. The SF-36 Physical Component Score (PCS) had a mean of 34.68 (SD = 7.57), which is below the normative mean of 50, suggesting poorer well-being. The Mental Component Score (MCS) mean was 40.65 (SD = 12.87), also below the normative value, indicating a range of mental health concerns, with some patients scoring particularly low. Overall, the questionnaires did not reveal a trend of somatization among our participants but did indicate mild symptoms of anxiety.

**Table 2.** Clinical questionnaires scores.

Patient num.	SDQ Total Score	BSI Somatization score	BSI general severity index score	HDES score	BIPQ Total Score	BIPQ open ended question	HADS Total Score	SF-36 PCS	SF-36 MCS
1	30	0.72	0.67	13.2	**63**	Root TX, Tension	**26**	**39.85**	**29.00**
2	22	0.28	−0.65	0.00	**60**	Dental TX, RT TMJ	**15**	**32.49**	53.63
3	23	2.66	**2.52**	3.20	**78**	Physicians’ mistake	**24**	**27.58**	**14.10**
4	28	0.39	0.22	18.93	**54**	Physicians’ mistake	**19**	**34.21**	54.46
5	**31**	1.37	0.26	2.10	**53**	Dentist, Fall	**25**	**29.28**	**35.48**
6	24	2.02	0.67	15.00	**58**	Genetics, Childhood Post Trauma, Physical Trauma	**22**	**20.00**	**42.32**
7	22	−0.37	0.19	1.07	**70**	Tension, Uncomfortable posture	**12**	**40.54**	53.36
8	22	−0.62	0.28	1.07	**51**	Dentist	**17**	**39.80**	**31.71**
9	27	0.28	−0.21	5.00	**52**	Don’t Know	9	**49.81**	51.65
10	22	−0.37	−0.51	4.28	**51**	Tumor	6	**32.14**	**43.21**
11	28	0.93	−0.04	3.92	47	Dental Implants, Occupational	5	**37.00**	**49.95**
12	**36**	0.50	−0.14	6.07	**63**	Explosion, Tooth Ache	**26**	**33.50**	**28.94**
Average	26.25	0.65	0.27	6.15	**58.33**	-	**17.17**	**34.68**	**40.65**
SD	4.54	0.98	0.82	6.15	9.00	-	7.76	7.57	12.87

*Major scores from the self-report questionnaires. Abnormal values are in bold. Cutoff values for clinical significance: BSI: Brief Symptom Inventory, Z-score >2 (abnormal); HADS: Hospital Anxiety and Depression Scale, total score >10 (suggestive of anxiety and/or depression); BIPQ: Brief Illness Perception Questionnaire, total score >42 (indicates a significant perceived threat) and >49 (a moderate threat); SDQ: Somatic Dissociation Questionnaire, total score >30 (suggestive of somatoform disorder); HDES: Hebrew Dissociative Experiences Scale, score ≥45–55 (indicative of dissociative psychopathology); SF-36: Short Form Health Survey, Physical (PCS) and Mental (MCS) Component Scores, mean = 50 (higher scores reflect better health status). SD: Standard Deviation; num: Number; TX: Therapy; RT: Right; TMJ: temporomandibular joint.*

### 3.3 Thematic analysis

Thematic analysis identified three overarching themes reflecting how patients perceive, interpret, and cope with PIFP:

(1) physical metaphors,

(2) emotional and cognitive reactions to pain, and

(3) encounters with the medical establishment.

#### 3.3.1 Theme 1: physical metaphors

Research has shown that chronic pain is most commonly described in terms of physical damage [17, 38]. Our thematic analysis has revealed several key themes related to the physical manifestations and the use of physical metaphors among patients with PIFP.

##### 3.3.1.1 Symbolic penetration

The first physical metaphor revolves around symbolic penetration, as participants expressed a fixation on dental procedures and described their pain with intrusive, penetrating, and gushing- out metaphors, often linked to a persistent “digging” or the opposite, a fear that something might “burst out” of their head or body. Exemplary quotes:

“*… he opened it and did something with those fine needles,”; “that he stirred something up there.*” (P1)

“*… as they dug in there and dug in there…. In the end, I said enough. Pull it out.*” (P7)

“*The pain is as if my jaw is about to burst out of me.*” (P3)

“*It started to crawl here… something from within that pushes out…*” (P5)

“*We’ll go into [the] facial pain—we’ll get it out. And I’ll tell you everything truthfully. Even if it doesn’t sit well with you.*” (P5)

##### 3.3.1.2 Facial pain analogous to emotional pain or a traumatic event

Seven of twelve participants alluded to a potential emotional correlation for their unusual facial pain. This hints at the possibility that PIFP might arise from emotional distress or latent traumas. During the interviews, several participants recounted experiences of emotional distress, culminating in fear of going insane, as well as past traumas, including feelings of anxiety, exposure, vulnerability, and stirring recollections of significant childhood events. Exemplary quotes:

“*Sometimes it’s like an exposed nerve.*” (P6)

“*[What] if it gets worse? How much [more] can I tolerate, I might go crazy…*” (P7)

“*And then something else happened [referring to past traumatic events], and then I said to myself—I’m not going to school.” “Even my parents used me a lot, but it’s not nice to say.”; “My parents only knew how to show me the horrors of humanity.*” (P5)

“*So, the mouth underwent trauma.*” (P8)

One of the participants even compared the facial pain to her holocaust experience:

“*…that it’s forbidden to cry, as if there’s nothing to cry about…. It was harder during the Holocaust.*” (P7)

The following quote from the interview with participant P9 demonstrates the physical proximity of pleasure with pain, describing the following:

“*I call it ‘until the next thrill’, until the next pain. [refers sarcastically to the anticipation of suffering from the anticipated painful episode]*”

##### 3.3.1.3 The pain as a silencer

Among six out of 12 participants, pain acted as a “silencer”. It left the patients with the feeling that they were unable to articulate themselves. The overwhelming intensity of the pain participants experienced hindered them from verbalizing their thoughts. Exemplary quotes:

“*I couldn’t speak, I simply couldn’t do anything…. I couldn’t open my mouth because the pain was very, very, very intense.*” (P1)

“*One must suffer in silence… I suffered in silence.*” (P7)

“*It’s forbidden to scream when it hurts, perhaps it’s allowed to cry… I had to scream but I’m not screaming… …a feeling like someone is clenching my neck.*” (P9)

“*A snake passing through my mouth.*” (P11)

Beyond the bodily metaphors used to describe their pain, participants also articulated complex emotional and cognitive reactions that shaped their overall experience of illness. This dimension is illustrated in the following theme.

#### 3.3.2 Theme 2: themes of emotional and cognitive reactions to pain

Both cognitive processes and cognitive content are believed to play crucial roles in adjusting to chronic pain [39]. Accordingly, participants in the study described their personal experiences as well as cognitive content related to managing chronic facial pain. Their descriptions highlighted the significant emotional and psychological toll their medical condition has taken on them, even to the point of life coming to a halt and imminent death.

##### 3.3.2.1 Catastrophic reaction

Cognitive content reflecting complex and catastrophic perceptions of facial pain emerged among 9 out of 12 participants. Exemplary quotes:

“*I felt that my life had stopped for a while…. It stops everything…… the pain dictates to me what I will do or not do.”* (P1)

“*Suddenly my life stopped… Now it stopped for me.*” (P3)

“*I got divorced, and I gave up on everything in life.”; “…And so I’m like stuck. Can’t move.*” (P5)

“*It’s frustrating, it’s where one stands aside and watches how their life passes by.*” (P6)

“*[I am] not functioning. Living-dead.*” (P8)

##### 3.3.2.2 Incomprehensibility

All participants (n = 12) encountered elements or experiences that resisted straightforward comprehension or interpretation. This theme highlights expressions of cognitive struggle, perplexity, and confusion in relation to various aspects of reality. Incomprehensibility manifested in multiple ways, with one notable example being the stark contrast between sections of highly detailed, specific content and others that were entirely incomprehensible, highlighting the participants’ inability to grasp or understand fully. Exemplary quotes:

“*Tooth number 46…, not sure if there is a filling or not… [it was] without a filling… Or was there a filling in it?*” (P1)

The patients describe an ongoing, endless pursuit for understanding and clarification of their condition, causing distress and a pervasive sense of incomprehensibility. Exemplary quotes:

“*It’s just that I want to understand further why it’s happening to me…. I want to know why it hurts me and no one gives me a solution, an answer, they don’t know, they guess.*” (P2)

“*It’s not triggered by something I do, it comes unexpectedly, it has no time, no place.*” (P2)

“*…. It could really be trauma or nerves or something… don’t know, it could be that God loves me, so He gave this to me.*” (P4)

“*What could it be? I’m in pain, I’m in pain all the time, it’s impossible to explain what this pain is, no one knows what this pain is.*” (P7)

“*…after all, I’ve been suffering from this for many years, [a pain] not understood.*” (P9)

“*What’s suddenly happening? Why is everything happening to me?*” (P11)

All these factors contribute to a pervasive sense of loneliness, a feeling of being misunderstood and unbelieved by those around them, and consequently, a deep sense of neglect and isolation. Exemplary quotes:

“*I’m just really alone, alone, alone… he [the doctor] sent me with the nurse, but I’m alone… I took care of my mother, and who will take care of me?*” (P2)

“*I always smiled, as if it were automatic.”; “No one really knows me.*” (P5)

“*Many people tell me, ‘But you look good…’ It annoys me terribly… So what if I look good? Does that mean I’m lying? I try very hard to keep to myself.*” (P6)

“*Really, today, I don’t need anyone in the world…*” (P7)

“*The family basically thinks I’m making the pain up.*” (P8)

“*It’s hard for her [mother] to hear that her daughter is sick.*” (P9)

“*In some parts of the process, I grappled with the dilemma of whether to rely on others.*” (P10)

There is a dysregulation of the proximity-distance axis in relationships. While many experience detachment and loneliness, some individuals tend to develop excessively symbiotic relationships with significant figures in their lives. This is manifested as clinging, where individuals become overly dependent on these connections for emotional support and validation, reflecting a complex interplay between seeking closeness and managing feelings of isolation:

“*They [parents] always controlled me in life… In the end, it turned out that they depend on me, not that I depend on them.*” (P5)

“*But it turns out that my mom, how can I say it? I don’t even know how to define it, but she instilled anxiety in me. The moment my husband returns to work, I need an ambulance.*” (P6)

“*I’m ‘a copy’ of my mom…by the way, one thing I forgot to mention is that my mom suffers from almost the same kind of pain….*” (P10)

“*My only pastime is visiting my mom… I go to visit her, sit with her, that’s where I’m most comfortable, because outside I’m uncomfortable, I have anxiety… So, at my mom’s, I know I’m sitting there comfortably… She also has issues…*” (P12)

##### 3.3.2.3 Loss of agency

Ten of 12 participants described their medical journey as occasionally passive, likening it to being “rolled” about, much like a leaf carried by the wind. This metaphor indicates a sense of diminished control or self-agency, suggesting that they felt as though external forces were propelling them, explicitly the painful experience, rather than actively directing their own path:

“*I hope I’ll come back…The pain sort of dictates what I should and shouldn’t do.*” (P1)

“*The nerve is alive, and each time it decides to hurt me?”; “I didn’t exist today.*” (P2)

“*The pain enjoys having a life of its own.*” (P3)

“*The pain keeps me at home…. And it deceives me.*” (P4)

“*A feeling like my brain is being grasped, like someone is really holding onto it with half a face…*” (P10)

Some of the patients exhibit tension between a feeling of control and a lack of control over the pain:

“*I know how to understand the pain and get it out… it will be okay…”; In contrast—”Just get it out! I’m not really in control.”; “Can’t get it out of my mouth.*” (P5)

“*… I’m no longer in control of my body… It’s overpowering…. I’ve learned to live with it. It does attack me at certain moments.*” (P7)

##### 3.3.2.4 Disconnecting from the emotional aspect

Eight of 12 participants exhibited emotional disengagement from their experience of pain, sometimes articulating this detachment explicitly and other times implying it more subtly. This pattern suggests a tendency to distance themselves from the idea that emotional states could influence their perception of pain. Exemplary quotes:

“*[The doctor] said it could be emotional stress…saying it’s anxiety—I find that hard to accept.*” (P3)

“*Pain is a disease; pain is not psychological.*” (P4)

“*I remember a specific teacher who told me that he always looks at me during breaks, and I looked sad to him. And I told him I’m not sad. I just have a headache.*” (P10)

“*…the funniest thing was that I also had something they couldn’t figure out.*” (P9 referring to the pain with inappropriate affect).

At times, the emotional distancing sounds almost dissociative:

Describing release through hydrotherapy/Watsu: “*I began to float…*”; “*An experience beyond the physical body, I call it.*” (P6)

In addition to their internal emotional struggles, patients’ narratives also revealed tensions and frustrations in their encounters with the medical system, forming the third central theme. The following section illustrates how these interactions reflect patients’ sense of confusion, mistrust, and disappointment toward healthcare providers.

#### 3.3.3 Theme 3: encounters with the medical establishment

##### 3.3.3.1 Complex interactions with medical figures

A recurring theme in the data was the complex and often challenging interactions patients had with healthcare providers. Complex interaction, confusion, and perplexity were apparent in all study participants except one. Participants expressed a varied spectrum of emotions, including frustration, confusion, and a lack of trust in the medical system. They frequently felt that their pain was not taken seriously or that they were not understood. Exemplary quotes:

“*I have thoughts that if I hadn’t undergone the root canal treatment, none of this would have happened.*” (P1)

“*…One of the doctors told me, Look what she [a different dentist] did it to you… she simply damaged a nerve and the nerve cannot be fixed.*” (P2)

“*I just want a doctor to come and heal me.*” (P3)

“*Because the pain started with the first doctor. And the second simply continued ‘the job’.*” (P3)

“*And you probably know how wise doctors are, how wrong they are…*” (P4)

Speaking about a miscarriage: “*I think I lost it because of a dental injection.*” (P5)

“*I asked to be discharged and he [the physician] didn’t want to discharge me.*” (P5)

Speaking about a treatment attempt with steroids: “*Ultimately, no, to the contrary, [the] steroids finished me off…*” (P6)

“*It’s not just injustice, it’s medical negligence.*” (P10)

“*I cannot forget the neurologist in the ER who said to me, ‘Listen, you just need to go for walks, get some fresh air’, as I suffer from an attack.*” (P10)

##### 3.3.3.2 Confusion and perplexity with the medical establishment

This theme captures participants’ sense that the medical system in general is unable to provide clear answers or effective treatment. The patients struggle to comprehend the nature and rationale behind the medical procedures they have undergone. There is a prevailing sense of misunderstanding regarding the healthcare system and the various treatments provided. Exemplary quotes:

“*See another doctor who will mess with my head like all the doctors?*” (P4)

“*I don’t know, I go to pain clinics, they inject me with this, inject me with that, they do dry needling, they do this to me, I don’t know what, they insert such needles into my jaw…*” (P8)

“*I don’t know, how do I feel? Down, how can I feel? One says this, one says that, I don’t know who to believe anymore.*” (P8)

“*I think that they [the dentists] basically pulled teeth out because I had chin pain, maybe even teeth that didn’t need to be extracted.*” (P11)

“*I don’t know… no matter how many times I go to doctors, they just keep prescribing me relaxation pills, antidepressants, and anti-anxiety medications. I think all these pills have harmed my intestines.*” (P12)

“*The specialist… is no longer even willing to talk to me. The last time I was with him, he didn’t even speak to me.*” (P12)

## 4. Discussion

### 4.1 General

This study reveals profound insights into the experiences of patients with PIFP who pursued dental extractions as a form of pain relief. Despite the narrow inclusion criteria (post-teeth extraction cases), a heterogeneous cohort was observed, with a wide age range and varying pain durations and localizations. Nevertheless, the thematic analysis highlighted three primary themes: physical metaphors, emotional and cognitive reactions to pain, and encounters with the medical establishment. Each theme sheds light on the complexity of PIFP and its significant impact on patients’ lives. The results from the questionnaires indicate that most patients do not meet the criteria for severe somatoform disorders, and overall, the cohort exhibited mild to moderate psychological distress, moderate to severe perceived illness severity, and poorer physical and mental health.

### 4.2 Discussion of the main themes

In the following section, we elaborate on the main themes identified in the patients’ narratives, examining their symbolic and psychological meanings as well as their clinical relevance. These themes illuminate the subjective world of individuals with PIFP, providing a deeper understanding of how their experiences of pain, emotion, and medical encounters intertwine. Notably, the three themes identified in this study correspond to Engel’s biopsychosocial model: physical metaphors capture the biological dimension, emotional and cognitive reactions to pain represent the psychological dimension, and encounters with the medical establishment reflect the social and relational dimension of the disorder [40].

#### 4.2.1 Theme 1—physical metaphors

The use of physical metaphors among patients, such as symbolic penetration and pain as a silencer, underscores the profound and debilitating nature of their suffering. These metaphors illustrate how patients externalize and personify their pain, reflecting an attempt to make sense of their persistent and debilitating condition. For instance, describing pain as “muzzling” or “silent” points to a desperate effort to articulate an experience that eludes straightforward description. This aligns with previous research indicating that metaphors can help patients frame and communicate their experiences of chronic pain [17, 38]. This situation reflects a more profound irony: while the term “silencing” implies an absence of voice, it is paradoxically used by those who are struggling to find their voice. Thus, the term becomes a tool for expressing the very thing it describes—an inability to be heard or to communicate. It’s an example of how language can both convey and complicate the experiences of those who feel muted or oppressed.

These bodily metaphors also resonate with psychological constructs of trauma and dissociation. The imagery of symbolic penetration evokes a sense of psychic invasion and loss of physical boundaries, often observed in post-traumatic experiences. In contrast, pain as a silencer reflects the dissociative tendency to mute affect and disconnect from overwhelming emotional states. Together, they suggest that patients’ metaphoric expressions not only communicate pain but also embody defensive and fragmentary processes characteristic of traumatic suffering and bodily and emotional dissociation. It is essential to recognize that the deliberate choice and prominence of metaphors, such as those related to invasion and muting, may be influenced by local cultural narratives surrounding pain, suffering, and psychosomatic distress, suggesting a potential area for comparative cross-cultural research. In the Israeli context, differences between Jewish Oriental and Ashkenazi cultural traditions may subtly influence the somatic localization or verbal expression of distress and the establishment of medical trust. However, this study’s sample size precludes definitive conclusions.

#### 4.2.2 Theme 2—emotional and cognitive reactions to pain

Patients’ emotional and cognitive responses to their pain reveal a landscape fraught with catastrophic thinking, incomprehensibility, and a sense of loss of agency. The descriptions of pain leading to a “halt” in life and a feeling of being a “living-dead” reflect the profound psychological impact of PIFP, mirroring catastrophic cognitive patterns observed in other studies on chronic pain [41]. The dissociation between patients’ experiences and their understanding of them aligns with findings in other chronic pain conditions, where patients similarly grapple with the disparity between their lived experiences and the interpretation and management of their symptoms [39]. Despite these intense qualitative experiences, a noticeable discrepancy exists between the BSI and HDES questionnaire data and the catastrophic and chaotic findings observed qualitatively. This contrast is reminiscent of the classic “la belle indifférence” phenomenon often seen in conversion disorders [42]. More broadly, the themes of incomprehensibility and depersonalization of the self (“living-dead”) strongly overlap with core symptoms found in trauma- and stressor-related disorders, specifically derealization and depersonalization, which are often observed as coping mechanisms in the face of chronic, intractable stress and pain. Indeed, the perception of high illness severity and poorer well-being, as recorded by the BIPQ and SF-36, corresponds with the emotional and cognitive struggles described by patients. This emotional distancing and cognitive struggle underscore the need for psychological support as an integral part of pain management. Healthcare providers should also recognize that such perceptions may contribute to learned helplessness, exacerbate patients’ distress, and be associated with depressive symptoms [43]. Therefore, therapeutic interventions should aim to enhance patients’ autonomy and active involvement in their treatment process.

#### 4.2.3 Theme 3—encounters with the medical establishment

The interactions with healthcare providers were marked by frustration and confusion. Patients frequently felt misunderstood or inadequately treated, echoing findings from studies on patients with chronic pain who report dissatisfaction with the healthcare system [12, 13]. These descriptions point to a state of medical mistrust, in which repeated experiences of being dismissed or inadequately helped erode patients’ confidence in medical care [44]. Recognizing and addressing such mistrust is essential to improving engagement and adherence. The sense of medical negligence and perplexity with treatment protocols emphasizes the need for improved communication and a more comprehensive approach to diagnosing and managing PIFP, including education for patients and healthcare providers. The question of whether these themes represent the general PIFP population or are unique to this subgroup with a high disease burden is central to interpretation. We hypothesize that the themes are significantly amplified by the experience of unnecessary dental extractions, as such interventions, viewed as potential cures, generate high levels of unmet expectations and accordingly profuse frustration and disappointment [45]. Moreover, ethically, the findings highlight the dilemmas clinicians face when balancing empathy and the wish to relieve suffering against the duty to avoid unnecessary or potentially harmful interventions. These findings, particularly regarding medical negligence and systematic failure, should be directly addressed by healthcare policymakers to institute mandatory, interdisciplinary training for dental and medical professionals on managing medically unexplained pain.

### 4.3 Clinical implications

The findings suggest several clinical implications. Recognizing and addressing the symbolic and metaphorical aspects of pain could enhance patient-provider communication and treatment efficacy [17, 20]. A holistic approach that considers the emotional and psychological dimensions of PIFP is crucial [18]. Multidisciplinary care models, involving pain specialists, psychologists, and dental professionals, may offer more integrated solutions [46]. Additionally, the frustration with the medical establishment underscores the need for more transparent and more empathetic communication, as well as patient education, as mentioned above. Ensuring patients are fully informed about their condition and treatment options could help reduce confusion and dissatisfaction.

While qualitative studies do not aim for statistical generalization, the themes identified in this study may offer transferable insights to similar contexts (*e.g.*, Burning Mouth Syndrome). The participants represent a distinct subgroup of PIFP patients—those who sought dental extractions as a means of pain relief. Understanding their experiences can inform clinicians encountering comparable clinical presentations in other chronic pain populations, where medical interventions are repeatedly pursued despite limited efficacy. These findings may, therefore, hold analytical generalizability, contributing to a broader understanding of the psychological and interpersonal dimensions of medically unexplained pain.

### 4.4 The influence of patient diversity

The sample’s demographic breadth, encompassing a wide age range (28–83 years) and both sexes (10 women, 2 men), provides a valuable, albeit complex, dimension to the findings. While chronic pain prevalence is higher in older adults, the inclusion of younger participants highlights the severe, life-interrupting nature of PIFP at any age, as reflected by the “catastrophic reaction” theme. Younger patients (*e.g.*, P1, 28-year-old student) may experience a greater perceived loss of agency and life path interruption, whereas older patients may present with more established, yet still perplexing, medical mistrust. Although the small number of male participants limits direct comparative analysis, pain research often indicates subtle differences in pain localization and expression between sexes. In this cohort, the most commonly reported pain sites—specific teeth and the jaw—were universal across the group (8 out of 12 patients), suggesting that the primary location of the medically targeted site (the teeth) overrides potential sex-based differences in pain reporting for this specific PIFP subgroup. Future, larger-scale studies should specifically examine whether men and women in the PIFP population exhibit differential pain location patterns and explore the psychosocial reasons for such variance.

### 4.5 Limitations and future research

This study’s limitations include a small sample size and the subjective nature of qualitative data, as well as a limitation of questionnaires based on self-reporting. Longitudinal studies exploring the long-term impact of dental extractions on PIFP and patient outcomes would provide further insights. Future research should also examine whether the themes identified here—such as somatization, desperation, and frustration with healthcare—are similarly present among patients who did not undergo dental extractions, and whether these psychological and behavioral patterns differ between the two groups.

Another limitation is the lack of data on systemic comorbidities such as depression, anxiety, and stress, smoking, and general health status, which may affect pain perception and coping. Although current psychiatric or psychological comorbidity was captured through the questionnaires, this information was not systematically verified. Future studies should include these variables to better characterize the biopsychosocial context of PIFP.

## 5. Conclusions

To conclude, this qualitative study sheds light on the intricate experiences of PIFP patients who sought dental extractions, revealing a complex interplay of physical, emotional, and cognitive factors. The identified themes underscore the multifaceted nature of PIFP and highlight the necessity for a comprehensive, multidisciplinary approach to treatment and support, including dentistry, pain medicine, neurology, psychiatry, psychology, and rehabilitation. Beyond the somatic dimension, the findings emphasize the importance of recognizing the psychic reactions, medical mistrust, and sense of helplessness often accompanying the disorder. Addressing these elements through integrative and empathic care may strengthen patients’ autonomy and engagement, improve adherence, and ultimately enhance therapeutic outcomes.
